# Gut microbiota in children with juvenile idiopathic arthritis: characteristics, biomarker identification, and usefulness in clinical prediction

**DOI:** 10.1186/s12864-020-6703-0

**Published:** 2020-04-07

**Authors:** Xubo Qian, Yong-Xin Liu, Xiaohong Ye, Wenjie Zheng, Shaoxia Lv, Miaojun Mo, Jinjing Lin, Wenqin Wang, Weihan Wang, Xianning Zhang, Meiping Lu

**Affiliations:** 1 Department of Rheumatology Immunology and Allergy, Children’s Hospital, Zhejiang University School of Medicine, Hangzhou, Zhejiang Province China; 2 State Key Laboratory of Plant Genomics, Institute of Genetics and Developmental Biology, Chinese Academy of Sciences, Beijing, China; 3 Department of Scientific Research Management and Medical Education, Jinhua Hospital of Traditional Chinese Medicine, Jinhua, Zhejiang Province China; 4 Department of Paediatric Rheumatology, The Second Affiliated Hospital and Yuying Children’s Hospital of Wenzhou Medical University, Wenzhou, Zhejiang Province China; 5 Nursing Department, Jiangnan Community Healthcare Center, Jinhua, Zhejiang Province China; 6 Department of Pediatrics, Wenling Maternal and Child Healthcare Hospital, Wenling, Zhejiang Province China; 7 Department of Pediatrics, Shaoxing People’s Hospital, Shaoxing Hospital, Zhejiang University School of Medicine, Shaoxing, Zhejiang Province China; 8 Department of Rheumatology Immunology, Jinhua Municipal People’s Hospital, Jinhua, Zhejiang Province China; 9 Department of Genetics, Institute of Genetics, Institute of Cell Biology, Zhejiang University School of Medicine, Hangzhou, Zhejiang Province China

**Keywords:** Juvenile idiopathic arthritis, Microbiota, Short-chain fatty acids, Butyrate, Propionate, Biomarker, Machine learning, Random forest model, Decision curve analysis

## Abstract

**Background:**

Recent studies have suggested that the gut microbiota is altered in children with juvenile idiopathic arthritis (JIA). However, age, sex, and body mass index (BMI) were not matched in the previous studies, and the results are inconsistent. We conducted an age-, sex-, and BMI-matched cross-sectional study to characterize the gut microbiota in children with JIA, and evaluate its potential in clinical prediction.

**Methods:**

A total of 40 patients with JIA and 42 healthy controls, ranging from 1 to 16 years, were enrolled in this study. Fecal samples were collected for 16S rDNA sequencing. The data were analyzed using QIIME software and R packages. Specifically, the random forest model was used to identify biomarkers, and the receiver operating characteristic curve and the decision curve analysis were used to evaluate model performance.

**Results:**

A total of 39 fecal samples from patients with JIA, and 42 fecal samples from healthy controls were sequenced successfully. The Chao 1 and Shannon–Wiener index in the JIA group were significantly lower than those in the control group, and the Bray-Curtis dissimilarity also differed significantly between the two groups. The relative abundance of 4 genera, *Anaerostipes*, *Dialister*, *Lachnospira*, and *Roseburia*, decreased significantly in the JIA group compared to those in the control group. The 4 genera included microbes that produce short-chain fatty acids (SCFAs) and were negatively correlated with some rheumatic indices. Moreover, 12 genera were identified as potential biomarkers by using the nested cross-validation function of the random forest. A random forest model constructed using these genera was able to differentiate the patients with JIA from the healthy controls, and the area under the receiver operating characteristic curve was 0.7975. The decision curve analysis indicated that the model had usefulness in clinical practice.

**Conclusions:**

The gut microbiota in patients with JIA is altered and characterized by a decreased abundance of 4 SCFA-producing genera. The decreases in the 4 genera correlated with more serious clinical indices. Twelve genera could be used as biomarkers and predictors in clinical practice.

**Trial registration:**

The study is registered online at the Chinese Clinical Trial Registry on 11 May 2018 (registration number: ChiCTR1800016110).

## Background

Juvenile idiopathic arthritis (JIA) is the most common rheumatic disease in children and one of the more common chronic illnesses of childhood [1]. JIA represents a heterogeneous group of disorders, all of which share the clinical manifestation of arthritis. The worldwide incidence of JIA ranges from 0.8–22.6/100,000 children per year, with the prevalence rate ranging from 7 to 401/100,000 children per year [1–3]. JIA is the most common cause of joint disability and vision loss, and leads to a decreased health-related quality of life, impaired social functioning, and increased medical expenses [4–7].

Although the etiology and pathogenesis of JIA are not completely understood, immunogenetic susceptibility and an external trigger are considered as the main risk factors contributing to JIA [8–10]. Studies on twins have shown that the concordance rates for JIA among monozygotic twins range from 25 to 40% [11], which suggests that non-coding factors, including epigenetics, female sex, and environmental factors, play an important role in the pathogenesis of JIA. Environmental factors, such as tobacco exposure, infectious agents, vitamin D deficiency, and the gut microbiota, not only trigger the development of rheumatic diseases, but are also involved in the transition from the preclinical to clinical stage [12–15]. Accordingly, environmental factors are pivotal in the development and progression of JIA.

Of the environmental factors, the gut microbiota has been implicated in the pathogenesis of JIA [16–20], rheumatoid arthritis [21], and metabolic diseases [22]. For example, the gut microbiota in these patients is altered [16–21], some of which are considerably correlated with clinical indices in patients with rheumatoid arthritis, such as anti-citrullinated protein antibody, rheumatoid factor, and C-reactive protein [21]. Furthermore, the random forest models constructed using the microbiota are able to differentiate rheumatic patients from healthy controls [20, 21]. Additionally, some microbe components are detected in synovial fluid [23] and liver tissue [24], which can trigger autoimmune responses [23, 24]. Finally, the causal relationships among the gut microbiome, short-chain fatty acids (SCFAs), and metabolic diseases have been demonstrated [22]. However, age, sex, and body mass index (BMI), which are the confounding factors that impact the composition of the gut microbiota [25–27], were not matched in some studies, and the results are inconsistent [17–20]. Whether the SCFA-producing genera dominate the differences between patients with JIA and healthy controls has not been well defined [17–20]. Furthermore, the clinical usefulness of the random forest models remains unclear [20, 21].

In order to address these problems, at least in part, we conducted an age-, sex-, BMI-, and ethnicity-matched cross-sectional study in Han Chinese children. We characterized the gut microbiota in patients with JIA, identified biomarkers, constructed a random forest model as a disease classifier using these biomarkers, and evaluated its usefulness in clinical prediction.

## Results

### Clinical and laboratory characteristics of the participants

In total, 40 children with JIA and 42 healthy children (HC) were enrolled into the JIA group and the control group, respectively. The median ages in the JIA group and the control group were 10.27 years and 9.95 years, respectively. There were no statistical differences in age, sex, and BMI between the two groups (Table 1). The disease subtypes, activity parameters, and other clinical indices are also shown in Table 1 and Additional file 2: Table S1. Five children with JIA did not have cytokine data (Additional file 2: Table S1).

**Table 1:** Demographic and clinical characteristics of the two groups

Characteristics	JIA group (*n* = 40)	Control group (*n* = 42)	Statistic	*P*-value
Age, median (IQR)	10.27 (3.09–11.56)	9.95 (3.20–11.60)	W = 827	0.907
Female	20	20	χ^2^ = 0	1.000
BMI, median (IQR)	16.23 (15.12–18.30)	16.60 (15.80–18.10)	W = 759	0.455
Disease duration, months, mean (SD)	3.47 (1.45)			
Subtypes of JIA				
Oligoarthritis, n (%)	17 (42.50)			
Polyarthritis, n (%)	9 (22.50)			
Enthesitis-related arthritis, n (%)	14 (35.00)			
Disease activity parameters				
cJADAS10, median (IQR)	9 (7–13)			
ESR, median (IQR)	20.50 (10.50–36.00)			
CRP, median (IQR)	3.00 (0.50–10.01)			
Autoantibody status				
ANA, median (IQR)^a^	0.00 (0.00–4.60)			
ACPA positive, n (%)	3 (7.50)			
RF positive, n (%)	3 (7.50)			
Cytokines				
IL-2, mean (SD), pg/ml	2.64 (1.07)			
IL-4, median (IQR), pg/ml	2.10 (1.30–2.07)			
IL-6, median (IQR), pg/ml	6.80 (2.85–16.70)			
IL-10, median (IQR), pg/ml	2.90 (2.15–3.90)			
TNF, median (IQR), pg/ml	2.00 (1.15–2.40)			
IFN-γ, median (IQR), pg/ml	3.50 (1.65–5.20)			
Cluster of differentiation				
CD3, mean (SD), %	70.60 (8.14)			
CD4, mean (SD), %	34.89 (6.94)			
CD8, mean (SD), %	29.58 (7.70)			
CD19, median (IQR), %	15.47 (11.04–17.64)			
CD3-CD16 + CD56+, median (IQR), %	10.47 (6.64–13.80)			
CD4/CD8, median (IQR)	1.14 (0.88–1.61)			

*ACPA* Anti-citrullinated protein antibodies, *ANA* Antinuclear antibody, *BMI* Body mass index, *CD* Cluster of differentiation, *cJADAS10* Juvenile arthritis disease activity score 10, *CRP* C-reactive protein, *ESR* Erythrocyte sedimentation rate, *IFN* Interferon, *IL* Interleukin, *IQR* Interquartile range, *RF* Rheumatoid factor, *TNF* Tumor necrosis factor

^a^Log10 transformed

### Gut microbiota diversities differed between the JIA and control groups

**Fig. 1:**
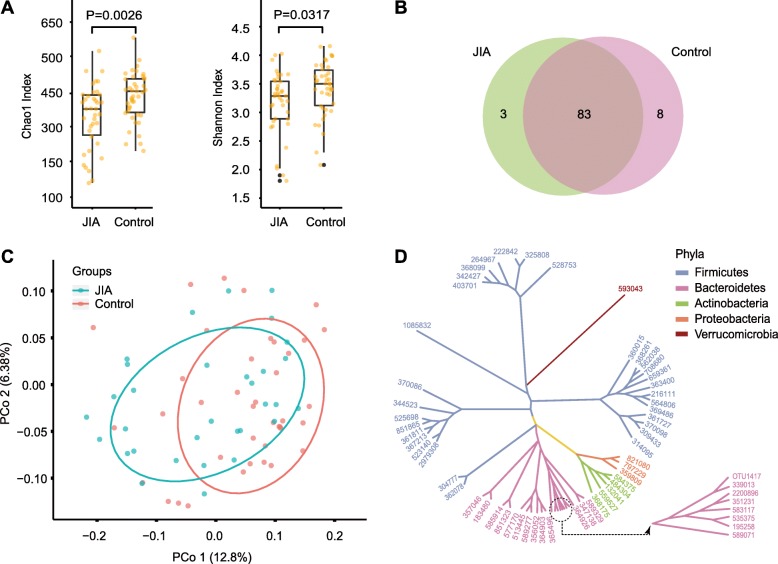
Diversity analyses show that the differences in the α- and β-diversities of the gut microbiota differ between the JIA and the control groups. **a** Comparisons of the Chao 1 and Shannon indices between the two groups. The two indices were significantly reduced in the JIA group compared to the control group (*P* = 0.0026 and 0.031, Wilcoxon test). **b** Venn diagram based on genera. The two groups have 83 shared genera, with 3 unique genera in the JIA group and 8 unique genera in the control group. **c** Ordination plot for the first two PCoA axes based on Bray-Curtis dissimilarity. The samples of the JIA and control groups are relatively clustered together, indicating that the Bray-Curtis dissimilarity differs between the two groups (*P* = 0.019, PERMANOVA test). **d** The phylogenetic tree was built using the OTUs greater than 0.3% (Additional file 2: Table S6). The OTUs in the plot are colored by phyla

A total of 40 JIA stool samples and 42 HC stool samples were collected, and 16S rDNA sequencing was completed in June 2019. In total, 39 JIA and 42 HC stool samples were successfully sequenced, and 7347 operational taxonomic units (OTUs) were obtained after the removal of singletons. After OTUs less than 0.001% were filtered, the remaining OTUs were classified into 11 phyla, 19 classes, 32 orders, 58 families, and 94 genera (Additional file 2: Table S2 and Table S3). The Chao 1 and the Shannon indices, two commonly used α-diversity indices, differed significantly between the two groups (*P* = 0.0026 and 0.031, Wilcoxon test; Fig. 1a, Additional file 2: Table S4); however, there was no significant difference in the Simpson index between the two groups (*P* = 0.248, Wilcoxon test; Additional file 1: Figure S3, Additional file 2: Table S4). The JIA and control groups had 3 and 8 unique genera, respectively; the two groups shared 83 genera (Fig. 1b, Additional file 2: Table S5). The Bray-Curtis dissimilarity, a commonly used β-diversity index, differed between the two groups (*P* = 0.019, R^2^ = 0.021, permutational multivariate analysis of variance [PERMANOVA] with 1000 Monte Carlo simulations; Fig. 1c). The phylogenetic tree, which was built using OTUs greater than 0.3%, showed that these OTUs belonged to the following five phyla: Firmicutes, Bacteroidetes, Actinobacteria, Proteobacteria, and Verrucomicrobia (Fig. 1d, Additional file 2: Table S6). The power of the study was 0.89 (Dirichlet-Multinomial Model with 1000 Monte Carlo simulations).

### Alterations of the gut microbiota in JIA patients and its associations with clinical indices

**Fig. 2:**
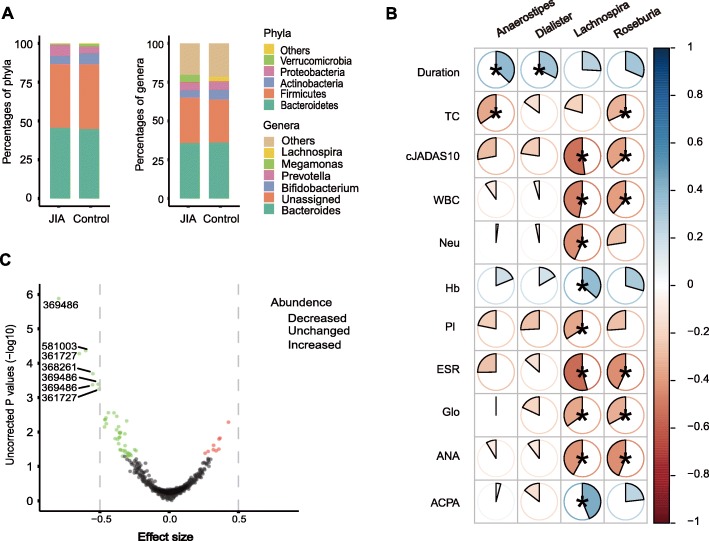
The compositional differences at phylum, genus, and OTU levels, and associations between genera and clinical indices. **a** The compositional differences of the phyla and genera between the two groups. **b** Associations between the relative abundance of the 4 genera and clinical indices. A pie chart with an asterisk indicates that the correlation coefficient reached significance after the *P*-value was adjusted. **c** Volcano plot of the OTUs. Green and red points represent the sample of those with *P*-values < 0.05 by Wilcoxon test (unadjusted *P*-values). The green and red colors indicate a decrease and increase in abundance, respectively. The effect size is the ratio of “the difference between groups” and “the maximum difference within groups.” In general, the effect size cut-off is more robust than *P*-values. The OTUs are considered biological markers if their absolute values of effect size are ≥0.5. Seven OTUs, marked with OTU numbers, have absolute values > 0.5, including the five OTUs identified by Wilcoxon test (Additional file 2: Table S8). ACPA: Anti-citrullinated protein antibody; ANA: Antinuclear antibody; cJADAS10: Clinical juvenile arthritis disease activity score 10; Duration: Disease duration; ESR: Erythrocyte sedimentation rate; Glo: Globulin; Hb: Hemoglobin; Pl: Platelet; TC: Total cholesterol; WBC: While blood cell; Neu: Neutrophil

At the phylum level, the most common phyla in the two groups were the Bacteroidetes, Firmicutes, Actinobacteria, and Proteobacteria (Fig. 2a). The Proteobacteria had higher abundance in JIA group (4.56%) as compared to that in the control group (4.03%), and the Verrucomicrobia was the opposite (0.0036% vs 0.048%); which were significantly different when analyzed using ALDEx2 package (*P* = 0.033, 0.029, respectively; Wilcoxon test). However, none reached significance when the *P*-values were adjusted for multiple testing corrections using the Benjamini–Hochberg method.

The genera of *Bacteroidetes*, *Bifidobacterium*, *Prevotella*, *Megamonas*, and *Lachnospira* were dominant in the two groups (Fig. 2a, Additional file 2: Table S3). The 4 genera *Anaerostipes*, *Dialister*, *Lachnospira*, and *Roseburia* had lower abundance in the JIA group (0.00, 0.011, 0.337, and 0.66%, respectively) as compared to those in the control group (0.040, 0.725, 2.244, and 1.162%, respectively), which reached significance when the *P*-values were adjusted for multiple testing corrections using the Benjamini–Hochberg method (Adjusted *P* = 0.031, 0.013, 0.041, and 0.011, respectively; Wilcoxon test; Additional file 2: Table S7). Of the 4 genera, *Dialister* was a genus of the Veillonellaceae family, the others belonged to the Lachnospiraceae family. All 4 genera belonged to the Clostridiales order and were found to be the microbes producing short-chain fatty acids (SCFAs) in previous studies [28–31]. No genera that were significantly enriched in the JIA group were identified by the Wilcoxon test (Adjusted *P* > 0.05; Additional file 2: Table S7). The 4 genera were significantly correlated with 11 clinical indices (all adjusted *P*-values < 0.05, “Holm” adjustment, Spearman’s correlation; Fig. 2b). Among the 4 genera, the *Lachnospira* and *Roseburia* were correlated with 9 and 6 clinical indices, respectively, while the *Anaerostipes* and *Dialister* were only correlated with 2 and 1 clinical indices, respectively. Out of the 18 associations with adjusted *P*-values < 0.05, 14 were negatively correlated and 4 were the opposite (Fig. 2b). The absolute values of the correlation coefficients ranged from 0.319 to 0.544 (Fig. 2b). The first three associations with greatest correlation coefficients were the ones between *Lachnospira* and ESR, WBC, and ANA.

At the OTU level, 55 OTUs were significantly different in abundance between the two groups when analyzed by Wilcoxon test (Fig. 2c, Additional file 2: Table S8); however, only 5 OTUs, labeled as OTU_361727, OTU_368261, OTU_369429, OTU_581003, OTU_470382, and, differed between the two groups after the *P*-values were adjusted (corrected *P* = 0.002, 0.015, 0.017, 0.037, and 0.043, respectively; the Benjamini–Hochberg method; Additional file 2: Table S8). The 5 OTUs had lower abundance in the JIA group (0.004, 0.004, 0.000, 0.000, and 0.010%, respectively) than those in the controls (0.158, 0.093, 0.132, 0.015, and 0.656%, respectively). No OTUs with increased relative abundance were identified by the Wilcoxon test after the *P*-values were adjusted.

### Twelve genera have the potential to serve as biomarkers in JIA diagnosis

**Fig. 3:**
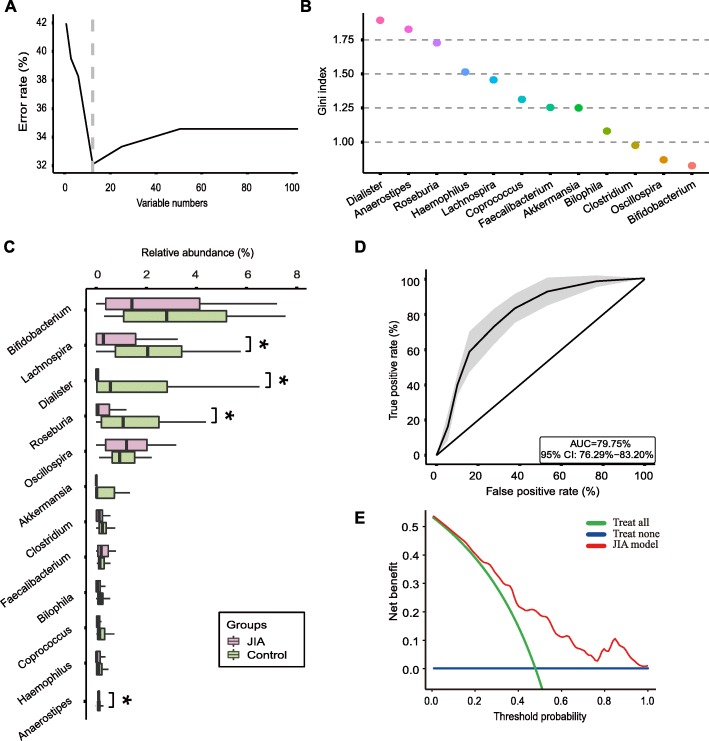
The random forest model constructed using 12 genera can be used as a disease classifier to differentiate JIA patients from healthy controls. **a** Plot of genera numbers vs error rates. As the genera numbers increased, the error rates decreased sharply. The dashed gray line marks the optimal cut-off for biomarker selection. This analysis indicated that 12 was the optimal predictor (genus) number. **b** The variable importance of the genera analyzed using the randomForest package in R. The most important 12 genera are listed in the plot. The greater the Gini indices, the more important the variables are. **c** The relative abundance of the 12 genera identified by the random forest model and Wilcoxon test. The 4 genera marked with an asterisk differed significantly in abundance between the two groups by Wilcoxon test (corrected *P* < 0.05). **d** ROC of the random forest model constructed using the 12 genera. The diagonal line in the graph marks an AUC of 0.5. The 95% confidence intervals are shown as shaded areas. **e** DCA for the random forest model constructed using the 12 genera. The y-axis measures the net benefit. The green line represents the situation with the assumption that all children received treatment due to JIA. The blue line indicates the net benefit under the assumption that no children received treatment due to JIA (e.g., representing the natural disease course without medical intervention so that the net benefit is constantly zero). The red line is above the green and blue lines, especially within the threshold probability of 0.23–0.77, which implies that the prediction model is able to achieve a greater net benefit than the situation when the children are treated or untreated without any model

To explore whether the gut microbiota can be used as biomarkers to differentiate JIA patients from healthy controls, we constructed six random forest models using all microbiota members at the phylum, class, order, family, genus, and OTU levels (Additional file 1: Figure S4). The model constructed using the microbiota at the genus level showed the best predictive accuracy of 67.9% (e.g., the out-of-bag error rate was 32.1%) among all taxonomic levels (Additional file 1: Figure S4). The results of the ten-fold nested cross-validation showed that as the predictors (e.g., variable or genus numbers in this case) increased, the out-of-bag error rate decreased sharply. When the genus number exceeded 12, the error rates no longer decreased (Fig. 3a). It indicated that the optimal number of biomarkers (genera) was 12. The 12 genera with highest variable importance are shown in Fig. 3b. Of the 12 genera, 10 had a lower abundance in the JIA group compared to the controls, while the other 2 genera (*Faecalibacterium* and *Oscillospira*) were the opposite (Fig. 3c). The 12 genera identified by the random forest method included the 4 genera that were identified by Wilcoxon test (Fig. 3c); at the OTU level, the two analysis methods identified similar results (Additional file 1: Figure S5).

After the 12 genera (biomarkers) were identified, we constructed a new random forest model as a disease classifier using these genera (Additional file 1: Figure S2). The out-of-bag error rate of the new model was 33.30%, which was only slightly higher than that of the model constructed using all microbiota members at the genus level. The area under the receiver operating characteristic curve (AUC) was 0.7975 (Fig. 3d). We used another tool, known as decision curve analysis (DCA), to evaluate the new model; the results of which showed that the net benefit obtained by the model was greater than the “treat-all” and “treat-none” curves within the threshold probability between 0.23 and 0.77 (Fig. 3e).

## Discussion

With the rapid development of sequencing technologies [32–35] and bioinformatics analysis methods [36, 37], the associations between microbiome and diseases have been demonstrated in recent years. The gut microbiota was found to be associated with rheumatic diseases approximately 50 years ago [38], and in the last 10 years, the involvement of the gut microbiota in the development and progression of JIA and other rheumatic diseases has been further established [24, 39]. In the current study, we found that the abundance of the 4 genera, *Anaerostipes*, *Dialister*, *Lachnospira*, and *Roseburia,* decreased in JIA patients, which were found to be the SCFA-producing microbes in previous studies [28–31]. The decreases in the 4 genera were correlated with more serious clinical indices. Moreover, we constructed a random forest model as a disease classifier using 12 biomarkers (genera), which was demonstrated that it had usefulness in clinical prediction.

Age is one of the main factors impacting the composition of the gut microbiota [25, 40], similar to sex [26], ethnicity [41], and others [42]. Thus, only age-, sex-, and ethnicity-matched healthy controls were selected in order to minimize confounding bias. Over the last decade, systemic JIA has increasingly been considered as an autoinflammatory condition, rather than an autoimmune disease. This distinguishes systemic JIA from other subtypes [43, 44]; thus, children with systemic JIA were not included in the current study.

The diversity in a single ecosystem or sample, called α-diversity, is often measured by the Chao1, Shannon–Wiener, and Simpson indices. The Chao1 and Shannon–Wiener indices give more weight to rare species, whereas the Simpson index puts more emphasis on common species [45]. In this study, the Chao1 and Shannon–Wiener index differed considerably between the two groups, but the Simpson index did not, implying that the rare species contributed to the differences to a greater extent than the common species. The results of the α-diversity analyses were similar to those of previous studies [17, 46]; however, other studies did not find any differences in the α-diversity indices [18–21].

Bray-Curtis dissimilarity, a commonly used β-diversity index, differed between the two groups, which indicated that the two groups had a different composition of gut microbiota. However, the R^2^ value was 0.02132, which showed that only 2.3% of the total variance could be explained by the group, e.g., JIA contributed to 2.3% alterations of the gut microbiota composition. The result of the β-diversity index was similar to those of previous studies [17, 20, 46, 47]. However, other studies either failed to find any differences in β-diversity indices or did not have relevant data [18, 19, 21]. The inconsistent α- and β-diversity results among these studies may be attributed to, at least in part, study design, study population, composition of JIA subtypes, duration of disease, and medication use prior to enrollment [39].

Diversity analyses have revealed that the two groups had a different composition of microbiota. Univariate community analysis further demonstrated the microbiota differences between the two groups, which were found to be the SCFA-producing microbes [28–31]. More specifically, at the genus level, the relative abundance of *Anaerostipes*, *Dialister*, *Lachnospira*, and *Roseburia* in JIA patients decreased significantly (corrected *P* < 0.05, Wilcoxon test), three of which are butyrate-producing microbes including *Anaerostipes* [28, 29], *Lachnospira* [30], and *Roseburia* [31]. The other genus, *Dialister*, is a propionate-producing microbe [48]. Compared to previous studies, decreases in the abundance of the *Anaerostipes* or *Lachnospira* were also observed in patients with JIA [18, 47], but these results were opposite to those observed in patients with rheumatoid arthritis [21, 46]. In contrast to the results in our study, the abundance of *Dialister* was increased in the previous study [49]. Hence, the findings of microbiota changes are inconsistent. Our study used the same sequencing method and similar data analysis methods as the previous studies. The key difference between them was the control selection. That is, whether the confounding factors of age, sex, BMI, and ethnicity were matched mainly contributed to the differences in the results between our study and others, because these confounding factors definitely impact the composition of gut microbiota [25–27, 40, 41]. Of these confounding factors, age probably is the main one, because the composition of gut microbiota in children younger than 16 years old varies substantially [25, 50, 51]. To some extent, other factors such as geography and diet may also have an effect on the differences [40, 52]. It has been demonstrated that SCFAs, including acetate, propionate, butyrate, and pentanoate, have considerable immunomodulatory effects through several pathways, such as inducing the differentiation of regulatory T cells, enhancing IL-10 production, and suppressing Th17 cells [53–55]. Moreover, butyrate administration suppressed the expression of inflammatory cytokines and ameliorated collagen-induced arthritis in mice [54]. Although causal relationships among gut microbiota, SCFAs, and metabolic diseases have been demonstrated previously [22, 56], these relationships remain vague in JIA and need to be further investigated.

Similar to a previous study [21], the correlations between the gut microbiota and some clinical indices were verified in the current study, but the absolute values of the correlation coefficients were relatively small. The 4 genera, especially the *Lachnospira* and *Roseburia*, were mainly negatively correlated with the clinical indices, e.g., when the relative abundance of the 4 genera decreased, the rheumatic clinical indices became higher or more active. This implies that, as in the previous study regarding metabolic diseases [22], the following sequential changes may be observed: Microbiota alterations (decrease in the abundance of SCFA-producing microbes), low concentrations of SCFAs, immune dysfunctions, and eventually rheumatic diseases.

The diagnosis of JIA primarily depends on medical history, physical findings, and the exclusion of other diseases, and is a challenging task in clinical practice. The 12 genera identified in our study were used to construct a new random forest model, which could help physicians to establish a diagnosis of JIA. The performance of a prediction model is usually evaluated with ROC, which assesses how well the predicted risks distinguish between patients with and without disease. Nevertheless, ROC cannot be used to evaluate whether a model could improve clinical decision making [57, 58]. In 2006, the DCA was introduced to overcome this limitation [59]. In our study, the AUC of the new model was 79.75%, which was lower than those reported in some earlier studies [20, 21], while the other studies did not report ROC results [17–19, 23, 46, 47]. The DCA analysis showed that our prediction model had greater clinical usefulness than the situation when the children were managed without a prediction model, particularly for those within the threshold probability of 0.23–0.77 [60]. However, the curve between the threshold probability of 0–0.23 is close to the green line (Fig. 3e), indicating that the additional gain of the model was not significantly different from the “treat-all” model where the threshold probability ranged from 0 to 0.23 [58]. To the best of our knowledge, our study is the first one to investigate the clinical usefulness of a random forest model based on the gut microbiota by using DCA. It remains unclear whether the prediction model can be further improved by integrating the clinical characteristics. Further studies of the model based on the gut microbiota are warranted to refine its application in clinical decision making.

There were several limitations to our study. First, oligoarthritis, polyarthritis, and enthesitis-related arthritis were lumped together in the JIA group. Second, the proportion of enthesitis-related arthritis among the subtypes was relatively high; thus, whether the results can be extrapolated to other study populations requires to be determined. Third, the sample size was relatively small due to the rigorous inclusion criteria; moreover, we did not conduct a longitudinal study. Lastly, we did not detect the SCFAs in fecal, blood, and synovial samples; thus, whether the decreased abundance of SCFA-producing genera leads to the concentration changes in these samples remains to be confirmed.

## Conclusions

In summary, this study shows that the gut microbiota is altered in patients with JIA, and is characterized by a decreased abundance of SCFA-producing genera, including *Anaerostipes*, *Dialister*, *Lachnospira*, and *Roseburia*. The 4 genera, especially the *Lachnospira* and *Roseburia*, were mainly negatively correlated with the clinical indices, e.g., when the relative abundance of the 4 genera decreased, the clinical indices became higher or more active. Furthermore, the random forest model constructed using 12 genera could accurately predict individuals with or without JIA, which indicates the 12 genera could be used as biomarkers and predictors in clinical practice. Further studies are warranted to explore the causal relationships among gut microbiota, SCFAs, and JIA, and to refine the model’s application in clinical decision making.

## Methods

### Study design, participants, and settings

We performed an age-, sex-, BMI-, and ethnicity-matched cross-sectional study at two tertiary hospitals between June 2018 and May 2019. All children were enrolled at the Children’s Hospital, Zhejiang University School of Medicine, and the Jinhua Municipal People’s Hospital, which are tertiary hospitals in Zhejiang Province, located in the southeastern part of China. The study had two groups: study group (JIA group) and control group (healthy controls). The flowchart of the study is shown in Additional file 1: Figure S1. The healthy children were those who visited their physicians for routine physical examinations at the two hospitals. The children with JIA met the following inclusion criteria: 1) Aged between 1 and 16 years old; and 2) new-onset JIA diagnosed according to the International League of Associations for Rheumatology classification criteria [61]. Specifically, new-onset was defined as disease duration between 6 weeks and 6 months, and absence of any treatment with disease-modifying anti-rheumatic drugs (DMARDs), biologic therapy, or steroids (ever). Healthy controls were age-, sex-, BMI-, and ethnicity-matched individuals with no history of JIA or the diseases listed in the exclusion criteria.

The exclusion criteria applied to both groups were as follows: 1) Children who were unwilling to participate in the study; 2) patients with systemic JIA; 3) individuals who had malnutrition [62] or were overweight [63]; 4) patients with recent (< 3 months prior) use of any antibiotics or probiotics; and 5) patients with co-morbidities including inflammatory bowel disease, allergic disorders, diabetes, primary immunodeficiency, tumors, or other chronic diseases.

### Sample collection and measurement methods

Specimens used in this study included fecal and blood samples. The former were used for 16S rDNA sequencing, and the latter were used to determine clinical indices such as complete blood cell count, biochemical profile, and serum cytokines. Fecal and blood samples from the JIA patients were collected within 24 h after the patients were admitted to the two hospitals, and fecal samples from the healthy controls were collected mainly at home. The fecal samples were stored at − 20 °C within 15 min after collection, and then were transferred to our laboratory on dry ice within 24 h of collection and stored at − 80 °C thereafter. Microbial DNA was extracted from the fecal samples using the TIANamp Stool DNA Kit (TIANGEN Biotech [Beijing] Co., Ltd., Beijing, China). A polymerase chain reaction was performed using 10–100 ng microbial DNA and 10 μM V3–V4 primers targeting 341F (CCTACGGGNGGCWGCAG) and 805R (GACTACHVGGGTATCTAATCC) and Phanta Max Master Mix (Vazyme Biotech Co., Ltd., Nanjing, China). Samples were pooled to equal concentrations, then sequenced on one lane of a MiSeq platform using the MiSeq Reagent Kit v3 (600 cycles; Illumina Inc., Shanghai, China), generating at least 30000 reads per sample.

Complete blood cell count was determined using a hematology analyzer (Mindray BC5310, Mindray Corp., China). Biochemical profile, such as alanine aminotransferase, aspartate aminotransferase, and creatinine, were determined using an automatic biochemical analyzer (HITACHI 7600, Hitachi Ltd., Japan). Serum cytokines including IL-2, IL-4, IL-6, IL-10, TNF-α, and IFN-γ were determined by the CBA Human Th1/Th2 Cytokine Kit II (BD Biosciences, USA) using a cell analyzer (BD FACSCanto™ II, Amersham Biosciences Corp., USA). Immunoglobulins were determined using a photometric assay analyzer (cobas® c 702 module, Roche Corp., Swiss). Autoantibodies, such as anti-citrullinated protein antibodies and antinuclear antibody, were determined using an immunofluorescence quantitative analyzer (HELIOS IOS-1000-AES, AESKU, Germany) and an automated western blot processor (Blotray-866, Rayto Life and Analytical Sciences Co., Ltd., China).

### Bioinformatics analysis

The QIIME pipeline [64] was used to process the sequencing data. All of the downstream analyses including diversity analyses and visualizations, differential abundance testing, correlation analysis, and biomarker identification and its evaluation were performed in R 3.6.1 (https://cran.r-project.org/).

### The processing of 16S rRNA sequencing data

The detailed methods are available in the literature [65]. We ran open-reference OTU picking at 97% identity, and then feature sequences were assigned to taxonomic classification using the classifier based on the Greengenes 13.5 database [66]. Samples with less than 1000 sequences per sample were considered failures and filtered out, and OTUs less than 0.001% were removed [42, 67].

#### α-Diversity measures and calculations

α-diversity indices, such as the Chao 1, Shannon–Wiener, Simpson, and Pielou’s evenness indices, were calculated using the vegan package 2.5–5 [68].

#### β-Diversity measures and comparisons

The Bray-Curtis dissimilarity, a commonly used β-diversity index, was used in our study, and was calculated using the distance() function (a function of R package was expressed as “function name()”) in the phyloseq package 1.26.1 [69]. The Bray-Curtis dissimilarity between the two groups was compared by the adonis() function in the vegan package [68]. In order to plot the figure of principal coordinate analysis (PCoA), three steps were carried out: 1) The zero values in the OTU table were replaced using the zCompositions package 1.3.2–1 [70]; 2) the data were transformed using the decostand() function (Hellinger transformation, from the vegan package) in order to alleviate the horseshoe effect [71]; 3) PCoA was performed using cmdscale() in the vegan package [68].

#### Visualization of the phylogenetic tree

The phylogenetic tree was plotted using the ggtree package 1.14.6 [72].

#### Differential abundance testing

The differential abundance testing was performed using the ALDEx2 package 1.16.0 [73]. The effect size and *P*-values, generated by this analysis, were used to draw a volcano plot, which was visualized with the ggplot2 package 3.2.1 [74].

#### Correlation analysis of the genera and the clinical indices

Spearman correlations between the genera and clinical indices were calculated using the psych package 1.8.12 [75], and visualized using the corrplot package 0.84 [76].

#### Identification of the genera serving as biomarkers

The built-in rfcv() function from the randomForest package 4.6–14 was used to explore the relationship between genera number and error rate [77, 78]. A nested cross-validation procedure was implemented in order to select an optimal predictor number using the rfcv(). The number corresponding to the minimum error rate was considered as an optimal predictor number or biomarker number. The OTU table at the genus level, which contained the selected biomarkers (biomarker data), was used for downstream analyses. The flowchart of the major steps involved in biomarker identification is shown in “Additional file 1: Figure S2”.

#### Construction of the receiver operating characteristic curve (ROC)

Ten-fold cross-prediction based on the biomarker data was performed to construct the ROC, i.e., the input samples (the biomarker data) were partitioned into 10 subsets, 9 subsets were used to fit random forest models in the randomForest package [78], and the rest subset was used to calculate prediction probability in the ROCR 1.0–7 [79]. This cross-prediction process was then repeated 10 times, with each of the 10 subsets used exactly once as the prediction data. The 10 sets of prediction results were used to calculate 95% confidence intervals and plot the ROC.

#### Decision curve analysis (DCA)

Two main steps were performed to plot the DCA curve: Step 1: a random forest model based on the biomarker data was fitted in the randomForest package [80], and step 2: votes, a probability matrix produced in random forest process, and sample metadata file were used to plot the DCA curve in the R function that is detailed in the reference [60]. The net benefit and threshold probability were calculated as previously described [57].

#### Sample size and power calculations

No formal sample size calculation was performed before the participants were enrolled.

The sample size was bigger than those used in previous studies [18, 19]. After the study was completed, the power of the study was calculated using the Dirichlet-Multinomial Model in the R package HMP [81].

### Statistical analysis

Continuous data were expressed as mean (standard deviation) and tested by Student’s *t* test or median with interquartile ranges and analyzed by Wilcoxon test. Categorical data were presented as percentages and were tested by Chi-square analysis or Fisher’s exact test. Spearman’s rank correlation was used to analyze correlations between non-normal data. A few children with JIA did not have cytokine data. These cytokine data were treated as missing values when performing correlation analysis in the R package psych [75]. All tests were two-sided tests, and *P* < 0.05 was considered statistically significant. *P*-values were adjusted for multiple testing using the “Benjamini-Hochberg” method or the “Holm” methods. All statistical analyses were performed with relevant packages in R.

## Supplementary information

**Additional file 1 **: **Figure S1**. Flowchart of the study. **Figure S2**. Flowchart of the major steps involved in biomarker identification. **Figure S3**. Plot of the Simpson and Pielou’s evenness indices. **Figure S4**. Predictive accuracies of different random forest models. **Figure S5**. The variable importance of OTUs.

**Additional file 2 **: **Table S1**. Mapping file (metadata); **Table S2**: OTU table; **Table S3**: Taxonomy of OTUs; **Table S4**. α diversity indices. **Table S5**. Data for Venn diagram. **Table S6**. OTUs greater than 0.3% of the total reads. **Table S7**. *P* values of genera (The results of compositional analysis using the ALDEx2 package at the genus level). **Table S8**. *P* values of OTU (The results of compositional analysis using the ALDEx2 package at the OTU level). **Table S9**. OTUs greater than 0.001% of the total reads.

## Data Availability

The 16S rDNA sequencing data are available in the NCBI SRA repository under BioProject PRJNA562467. The R codes and its input of the figures in the manuscript are available at https://github.com/qianxubo/microbiota-stage-1.

## Abbreviations

ACPA: Anti-citrullinated protein antibody
ANA: Antinuclear antibody
AUC: Area under receiver operating characteristic curve
BMI: Body mass index
CD: Cluster of differentiation
cJADAS10: Clinical juvenile arthritis disease activity score 10
CRP: C-reactive protein
DCA: Decision curve analysis
DMARDs: Disease-modifying anti-rheumatic drugs
ESR: Erythrocyte sedimentation rate
Glo: Globulin
Hb: Hemoglobin
HC: Healthy children
IFN: Interferon
IL: Interleukin
IQR: Interquartile range
JIA: Juvenile idiopathic arthritis
Neu: Neutrophil
OTUs: Operational taxonomic units
PCoA: Principal coordinate analysis
Pl: Platelet
RF: Rheumatoid factor
ROC: Receiver operating characteristic curve
SCFAs: Short-chain fatty acids
TC: Total cholesterol
TNF: Tumor necrosis factor
WBC: While blood cell
