# Supplementary material for: Gut microbiota in children with juvenile idiopathic arthritis: characteristics, biomarker identification, and usefulness in clinical prediction
Source: BMC Genomics. 2020 Apr 7;21:286. doi: 10.1186/s12864-020-6703-0 (PMC7137182; doi:10.1186/s12864-020-6703-0)
Supplement: Supplementary file 1 — Additional file 1 : Figure S1. Flowchart of the study. Figure S2. Flowchart of the major steps involved in biomarker identification. Figure S3. Plot of the Simpson and Pielou’s evenness indices. Figure S4. Predictive accuracies of different random forest models. Figure S5. The variable importance of OTUs. [file 12864_2020_6703_MOESM1_ESM.docx]

**Additional File 1 for**

**Gut Microbiota in Children with Juvenile Idiopathic Arthritis: Characteristics, Biomarker Identification, and Usefulness** **in Clinical Prediction**

Xubo Qian, Yong-Xin Liu, Xiaohong Ye, Wenjie Zheng, Shaoxia Lv, Miaojun Mo, Jinjing Lin, Wenqin Wang, Weihan Wang, Xianning Zhang and Meiping Lu

This file includes:

[Figure S1 Flowchart of the study 2](#_Toc30061517)

[Figure S2 Flowchart of the major steps involved in biomarker identification 3](#_Toc30061518)

[Figure S3 The plot of the Simpson and Pielou’s indices 4](#_Toc30061519)

[Figure S4 Predictive accuracies of different random forest models 4](#_Toc30061520)

[Figure S5 The variable importance of OTUs 5](#_Toc30061521)

Other additional files for this manuscript are available at: https://doi.org/10.1186/s12864-020-6703-0

# Figure S1 Flowchart of the study

Excluded (22) #

Study group (40)

62 children with JIA

Excluded (7) *

Blood samples

Fecal samples

Microbiota,

39 sequenced successfully

Comparisons: α and β-diversity indices, biomarkers

Control group (42)

49 healthy children

Microbiota,

42 sequenced successfully

Correlations

Laboratory results and other clinical indices

**Figure S1** Flowchart of the study. # Thirteen children with JIA did not meet the inclusion criteria, and nine refused to participate the study. * Seven healthy children did not meet the inclusion criteria.

# Figure S2 Flowchart of the major steps involved in biomarker identification

Construction of random forest models using all microbiota members at different taxonomic levels

Selection of a model with the best predictive accuracy

Identification of the optimal predictor number (χ) using rfcv() function

Selection of the “χ” predictors (12 genera in this study)

Construction of a new random forest model using “χ” predictors

Evaluation of the new model (disease classifier) using ROC

Evaluation of the new model (disease classifier) using DCA

**Step 1**

**Step 2**

**Step 3**

**Figure S2** Flowchart of the major steps involved in biomarker identification. Step 1 is to determine the optimal predictor number. Step 2 is to pick out the predictors, and to construct a new random forest model using these predictors. Step 3 is to evaluate the performance of the new random forest model using receiver operating characteristic curve (ROC) and decision curve analysis (DCA).

# Figure S3 The plot of the Simpson and Pielou’s indices


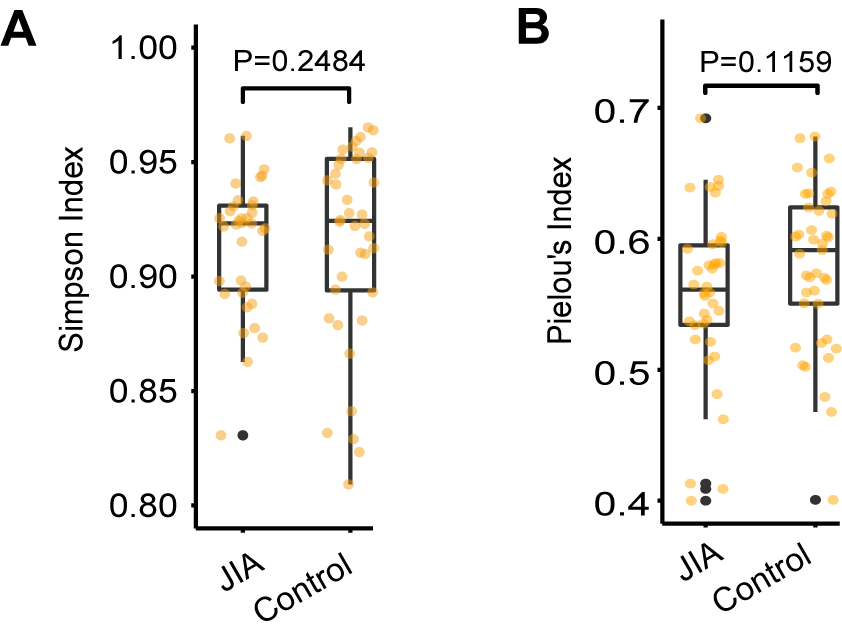


**Figure S3** Plot of the Simpson and Pielou’s evenness indices. These indices do not differ between the JIA group and the control group.

# Figure S4 Predictive accuracies of different random forest models


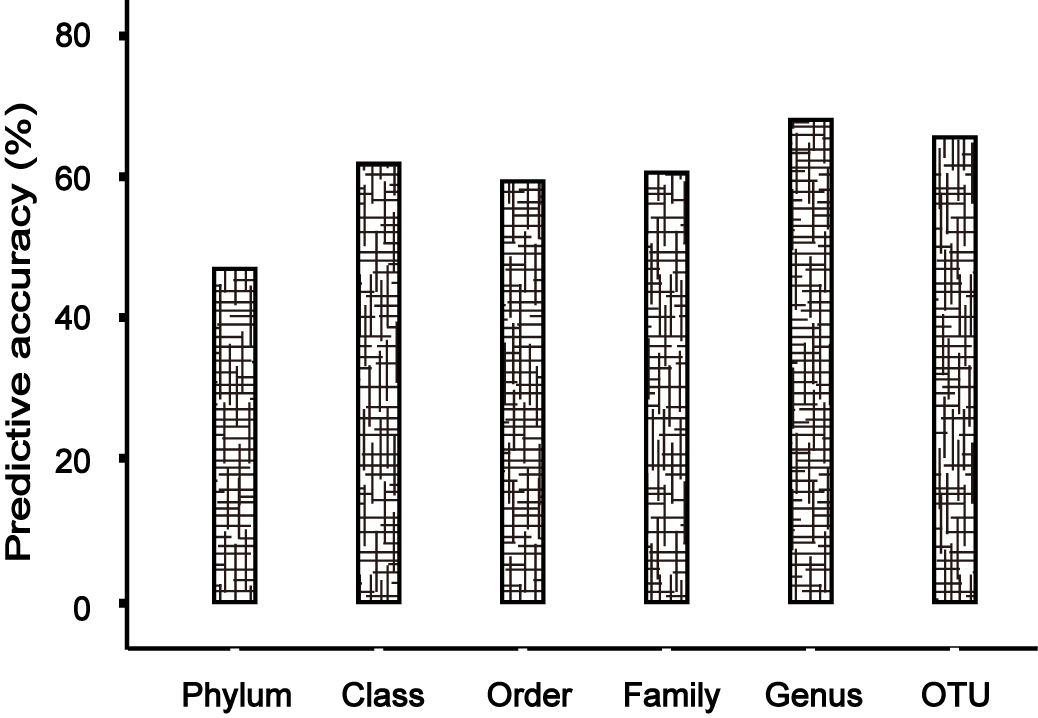


**Figure S4** Predictive accuracies of different random forest models constructed using all microbiota members at the phylum, class, order, family, and genus levels. The model constructed using the microbiota at the genus level shows a best predictive accuracy of 67.9%.

# Figure S5 The variable importance of OTUs


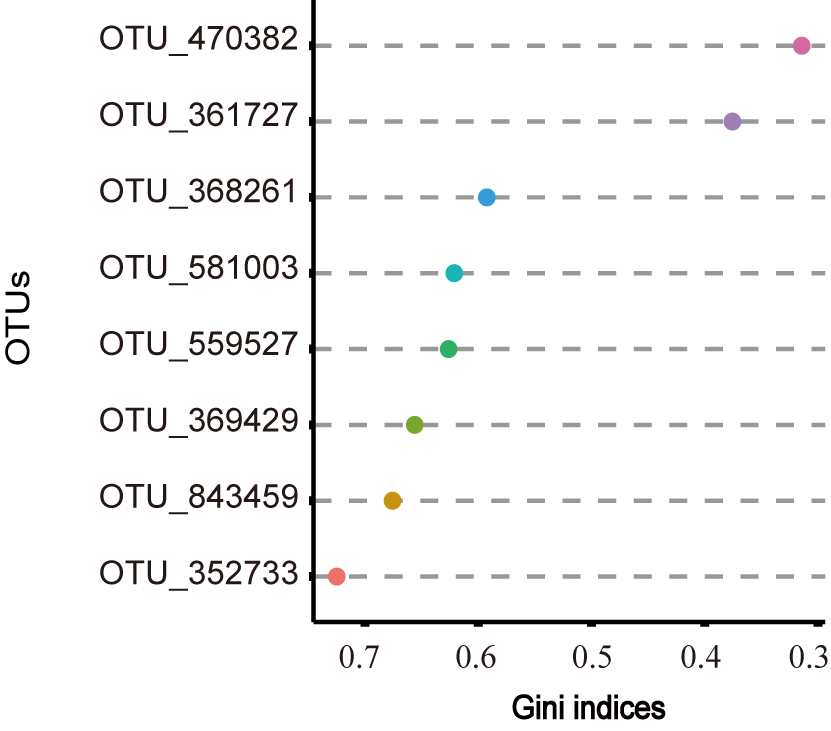


**Figure S5** The variable importance of OTUs analyzed using the random forest model. The greater the Gini indices, the more important the variables are. The abundance of OTU_361727, OTU_470382, OTU_369429, OTU_581003, and OTU_368261 decreased significantly in the JIA group compared with those in the control group by Wilcoxon test (corrected *P* < 0.05).
